# Heritability estimates of the novel trait ‘suppressed *in ovo* virus infection’ in honey bees (*Apis mellifera*)

**DOI:** 10.1038/s41598-020-71388-x

**Published:** 2020-08-31

**Authors:** Dirk C. de Graaf, Dries Laget, Lina De Smet, David Claeys Boúúaert, Marleen Brunain, Roel F. Veerkamp, Evert W. Brascamp

**Affiliations:** 1 Honeybee Valley, Ghent University, 9000 Ghent, Belgium; 2 Department of Biochemistry and Microbiology, Ghent University, 9000 Ghent, Belgium; 3 Wageningen University and Research, Animal Breeding & Genomics, 6708 PB Wageningen, The Netherlands

**Keywords:** Genetics, Diseases

## Abstract

Honey bees are under pressure due to abnormal high colony death rates, especially during the winter. The infestation by the *Varroa destructor* mite and the viruses that this ectoparasite transmits are generally considered as the bees’ most important biological threats. Almost all efforts to remedy this dual infection have so far focused on the control of the *Varroa* mite alone and not on the viruses it transmits. In the present study, the sanitary control of breeding queens was conducted on eggs taken from drone brood for 4 consecutive years (2015–2018). The screening was performed on the sideline of an ongoing breeding program, which allowed us to estimate the heritabilities of the virus status of the eggs. We used the term ‘suppressed *in ovo* virus infection’ (SOV) for this novel trait and found moderate heritabilities for the presence of several viruses simultaneously and for the presence of single viral species. Colonies that expressed the SOV trait seemed to be more resilient to virus infections as a whole with fewer and less severe Deformed wing virus infections in most developmental stages, especially in the male caste. The implementation of this novel trait into breeding programs is recommended.

## Introduction

The economic value of the honey bee can be almost entirely attributed to its pollination services for agricultural crops^1^. However, honey bees are under pressure due to abnormal high colony death rates, especially during the winter^2^. There are several possible explanations for the increased mortality, but the infestation by the *Varroa destructor* mite and the viruses that this ectoparasite transmits are generally considered the most important biotic threats^3,4^. The mite was found to be an efficient vector of viruses even to the extent that initially the clinical symptoms caused by the Deformed wing virus (DWV) were wrongly attributed to the mite^5^. The *Varroa* mite and DWV interact in many different ways, resulting in an increase in DWV virulence^6,7^ and colony mortality^8–12^. These effects are partially due to the mutualistic symbiotic relationship between both, in which the mite provides transmission of the virus when it feeds on the bee, whereas the virus undermines the immunity of the honey bee by interfering with NF-κB signaling, possibly facilitating the mite’s trophic activity^13^. Almost all efforts to remedy this dual infection have so far focused on the control of the *Varroa* mite alone. Indeed, several mite-control strategies have been set up in beekeeping practice that rely on acaricide medication or other treatments, biotechnical apicultural methods and selection for *Varroa* resistance^14^. The control of the viruses transmitted by the mite is therefore achieved almost exclusively indirectly, by limiting the severity of the mite infestation. One exception to this is the treatment with double-stranded RNA to provoke a targeted antiviral immune response based on RNA interference, but this is rarely used in beekeeping practice^15,16^.

The transmission of viruses between bees by the vectoring *Varroa* mite is described as horizontal transmission, as it is from one individual to another from the same generation by either direct or indirect contact. Virus transmission through bodily contact, trophallaxis, feeding and common flower visits are also categorized under horizontal transmission. On the other hand, vertical transmission refers to the passing of an infectious agent from parent to offspring via eggs and semen. It has been demonstrated that DWV vertical transmission occurs predominantly by virus particles adhering to the surface of the egg (transovum) rather than intracellularly^17^. Queens can become infected by trophallaxis and during natural mating^18^ or instrumental insemination^19^, and both the drone’s endophallus^18,20^ and semen^19,21^ can be the source of virus infection. In contrast to worker brood and drone brood, queen brood cells have an extraordinary low *Varroa* infestation level^22^, so it is unlikely that the queen becomes virus infected by vectoring. Virus infection of the queens can contribute to queen failure^18^, which seems to be an important cause of colony collapse^23,24^. The queen’s ovaries can become heavily infected^18,25^ leading to typical pathological reactions with discoloration^26^.

Because virus infection of a queen can influence her performances and represents an important source of infection for her progeny by vertical transmission, we started in 2012 a sanitary control of the breeding queens from Flemish beekeepers in the North of Belgium following a non-destructive approach. Freshly laid eggs taken from worker brood were examined for the commonly occurring bee viruses and led to the observation that vertical transmission occurs rather frequently^27^. Here we present the continuation of this study in which the sanitary control of the queens was conducted on eggs taken from drone brood for 4 consecutive years (2015–2018), before finally estimating the heritabilities of the virus status of the eggs. The term ‘suppressed *in ovo* virus infection’ (SOV) was given to this novel trait. We hypothesized that queens that were capable of clearing a visceral virus infection and consequently deposited virus-free eggs, would pass this predisposition of protective anti-viral immune responses on to their descendants. Drone brood eggs were our target samples as they are unfertilized and thus carry only the alleles of the breeding queen.

## Results

### Descriptive statistics

Fifty four Flemish queen breeders volunteered drone eggs to determine the virus status of the queens (from 2015 to 2018). The dataset contained 625 samples of 560 Carnica queens, 25 Buckfast queens and 23 queens of unknown subspecies (see Supplementary Table S1 online). Sixteen Carnica queens provided two samples and one queen three samples. The samples were analyzed for the presence of DWV, Acute bee paralysis virus (ABPV), Black queen cell virus (BQCV) and Sacbrood virus (SBV) and were scored 0 or 1 (0 = absence; 1 = presence) for each of the viruses and for total virus status (TVS). At the start (2015), the number of breeding queens involved in the sanitary screening was rather low (see Supplementary Fig. S1A online). This changed in 2016, when for the first time close to 200 queens were included. In the beginning, the vast majority of tested queens were bred from mother queens with an unknown virus status (UQ subgroup). But, the proportion of descendants of virus-negative queens (DV-Q subgroup) grew steadily and represented in 2018 almost 40% of the tested queens (see Supplementary Fig. S1A online).

**Figure 1:**
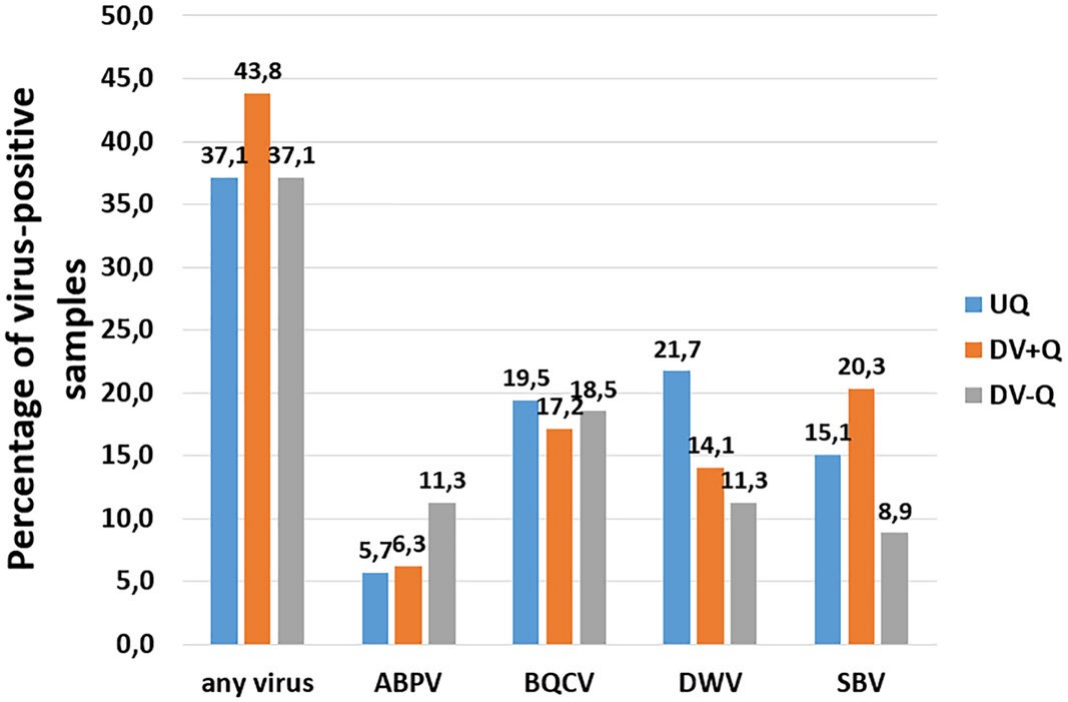
Percentage of virus-positive samples per subgroup. On the left, the percentage of samples with a positive total virus score (TVS, any virus) are given. On the right side of this, the scores for the separate virus species are given. Samples were categorized in three defined subgroups: descendants of virus-positive queens (DV + Q), descendants of virus-negative queens (DV-Q) and unspecified daughter queens (UQ).

The percentage of TVS-positive samples fluctuated slightly over the successive years (see Supplementary Fig. S1B online). And when the entire period 2015–2018 was taken in consideration, the percentage of virus-positive samples (any virus) differed slightly between the different subgroups (Fig. 1). However, for the virus species separately, some remarkable differences could be observed. The occurrence of DWV and SBV in eggs was considerably lower in the DV-Q subgroup as compared to the DV + Q (descendants of virus-positive queens) and UQ subgroups. That was not the case for ABPV and BQCV (Fig. 1). And we found the most ABPV, DWV and SBV, in the DV-Q, UQ and DV + Q subgroups, respectively.

**Figure 2:**
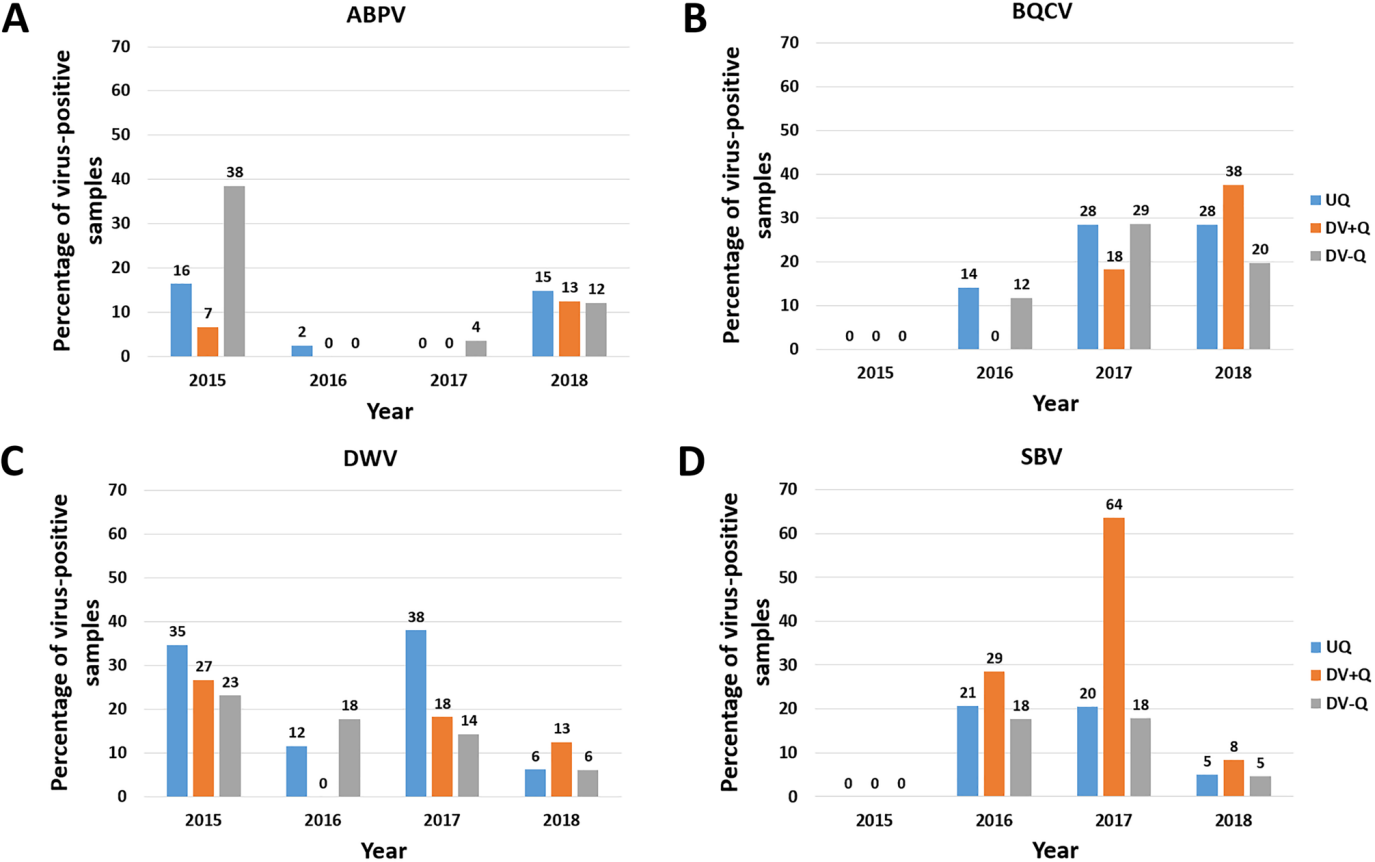
Percentage of ABPV- (in **A**), BQCV- (in **B**), DWV- (in **C**) and SBV-positive samples (in **D**) in successive years. The abbreviations of the three defined subgroups is explained under Fig. 1. The indicated values were rounded for readability.

When we look at the successive years, the DV-Q subgroup mostly scored better (lower) for DWV (except in 2016) and SBV and the occurrence of these viral species fell to its lowest level in 2018 with only 6.1 and 4.5% of the samples being positive, respectively (Fig. 2). On the contrary, no clear pattern could be found for ABPV and BQCV, though in 2018 the number of BQCV-positive samples was only half in DV-Q compared to DV + Q. Although these data have no burden of proof, they suggest that the DWV and SBV virus status of the daughter queens (as determined by the virus status of the drone eggs) can at least partially be explained by genetic factors. We substantiated this by the estimation of the heritabilities of the SOV trait and made a distinction between the form that offers protection against all tested virus species and against the individual virus species.

### Heritabilities

The estimation of heritabilities was restricted to drone eggs of 560 Carnica queens. The majority of these queens with their colonies were part of an ongoing international breeding program (Beebreed) with some 10,000 colonies annually. Table 1 summarizes the heritabilities estimated for the status of individual viruses and for TVS. We used a threshold model, assuming that the phenotypes had a binomial distribution, while the underlying genetic liability followed a normal distribution. We used the threshold model because the phenotypes were scored 0 and 1 and the trait incidence was low.

**Table 1:** Heritabilities for the different traits with standard errors between brackets and the statistical level of significance (*p* value) of environmental similarity of dams and daughters.

Trait/virus	Heritability	Significance of environmental similarity
SOV/ABPV	0.32 (0.16)	0.76
SOV/BQCV	0.53 (0.14)	0.97
SOV/DWV	0.21 (0.12)	0.92
SOV/SBV	0.33 (0.12)	0.56
SOV/TVS	0.25 (0.09)	0.50

The heritability for SOV/TVS was estimated to be 0.25. The estimated heritabilities for three individual viruses were of the same order of size. The estimate for SOV/BQCV was larger but due to the limited size of the dataset the standard errors of all estimates were high. Nevertheless it may be concluded that the different forms of SOV were moderately heritable.

### Environmental similarity of dams and daughters

The statistical model used to estimate the heritability also included the virus score of the dam. This is a fixed effect and represents the non-genetic (environmental) effect of the virus score of the dam on that of the daughter, as expressed in their respective drone eggs. The significance levels (*p*-values) for the fixed effect for queen’s virus loads were presented in Table 1. Various routes of this environmental similarity of dams and daughters might be envisaged like the direct transfer of virus from dams to daughters through eggs or various developmental stages. Also contamination in the internal environment as wax or royal jelly might be possible, or in the external environment as through the beekeeper or foraging. The statistical analysis did not confirm the relevance of those routes.

### Correlations

Ideally one would like to estimate genetic correlations between the different virus traits. In a dataset of this size that is not useful. Alternatively, phenotypic correlations were computed between the traits, albeit that trait variables were adjusted for the effects of the test location classes to which they belong. Table 2 summarizes these correlations between the traits. Generally speaking the correlations between individual viruses were low while SOV/BQCV and SOV/DWV correlated higher to SOV/TVS than the other two viruses.

**Table 2:** Correlations between the traits calculated from the deviations from fixed-effect-estimates**.** Standard errors are between brackets.

	SOV/BQCV	SOV/DWV	SOV/SBV	SOV/TVS
SOV/ABPV	0.17 (0.05)	0.04 (0.05)	0.15 (0.05)	0.33 (0.04)
SOV/BQCV		0.07 (0.05)	0.13 (0.05)	0.57 (0.03)
SOV/DWV			0.02 (0.05)	0.51 (0.03)
SOV/SBV				0.33 (0.04)

### Virus load of all developmental stages

**Figure 3:**
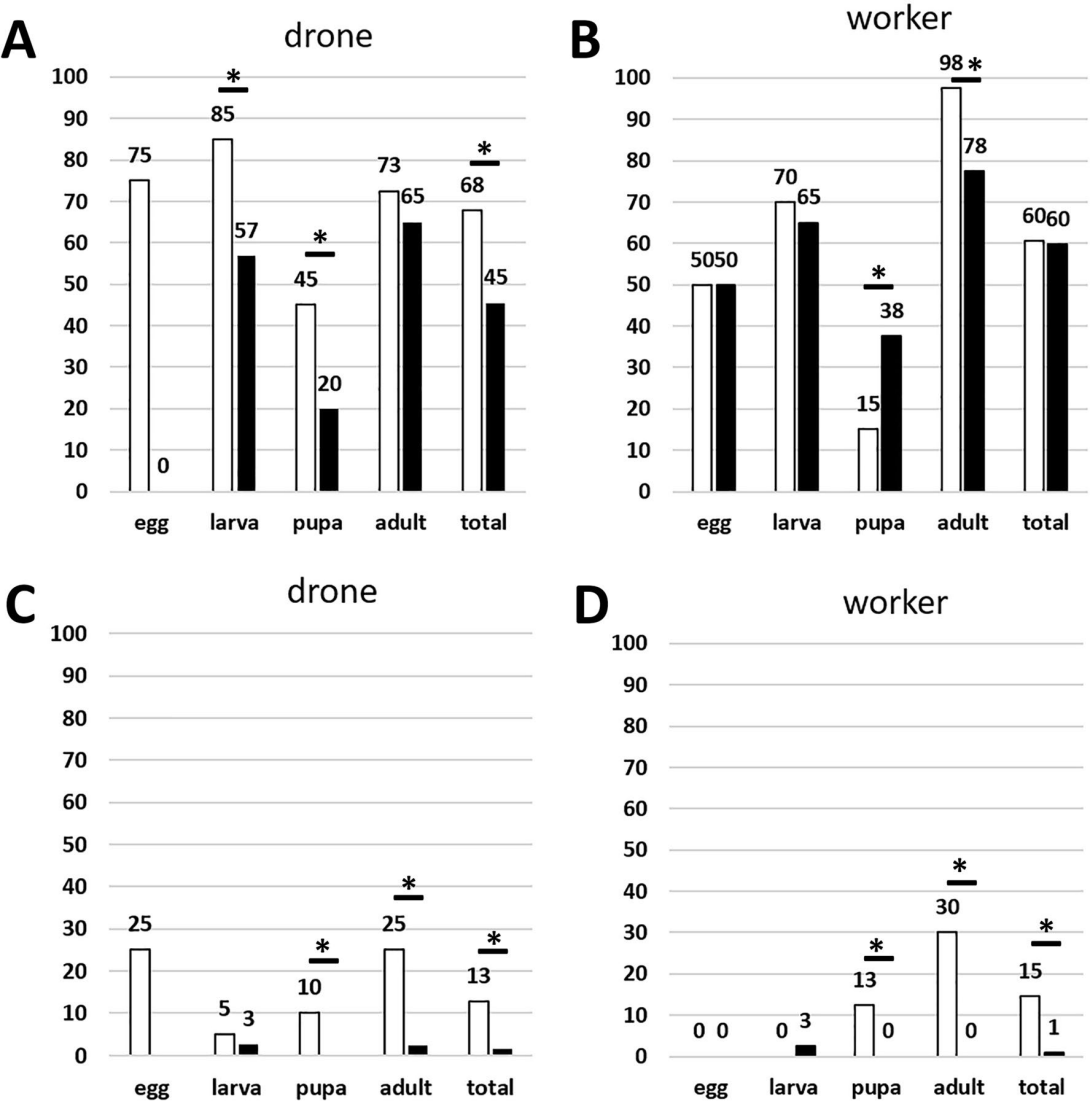
Percentage of DWV-positive samples across different developmental stages of drones (in **A**) and worker bees (in **B**) with the threshold value for a positive virus determination set at 10^5^ viral genomic copies per bee (or per 10 pooled eggs). White bars represent samples from the control group; black bars represent samples from the SOV group. In (**C**,**D**) the same graphs were shown with threshold value for a positive virus determination set at 10^9^ viral genomic copies per bee (or per 10 pooled eggs). Significant differences are indicated by an asterisk.

**Figure 4:**
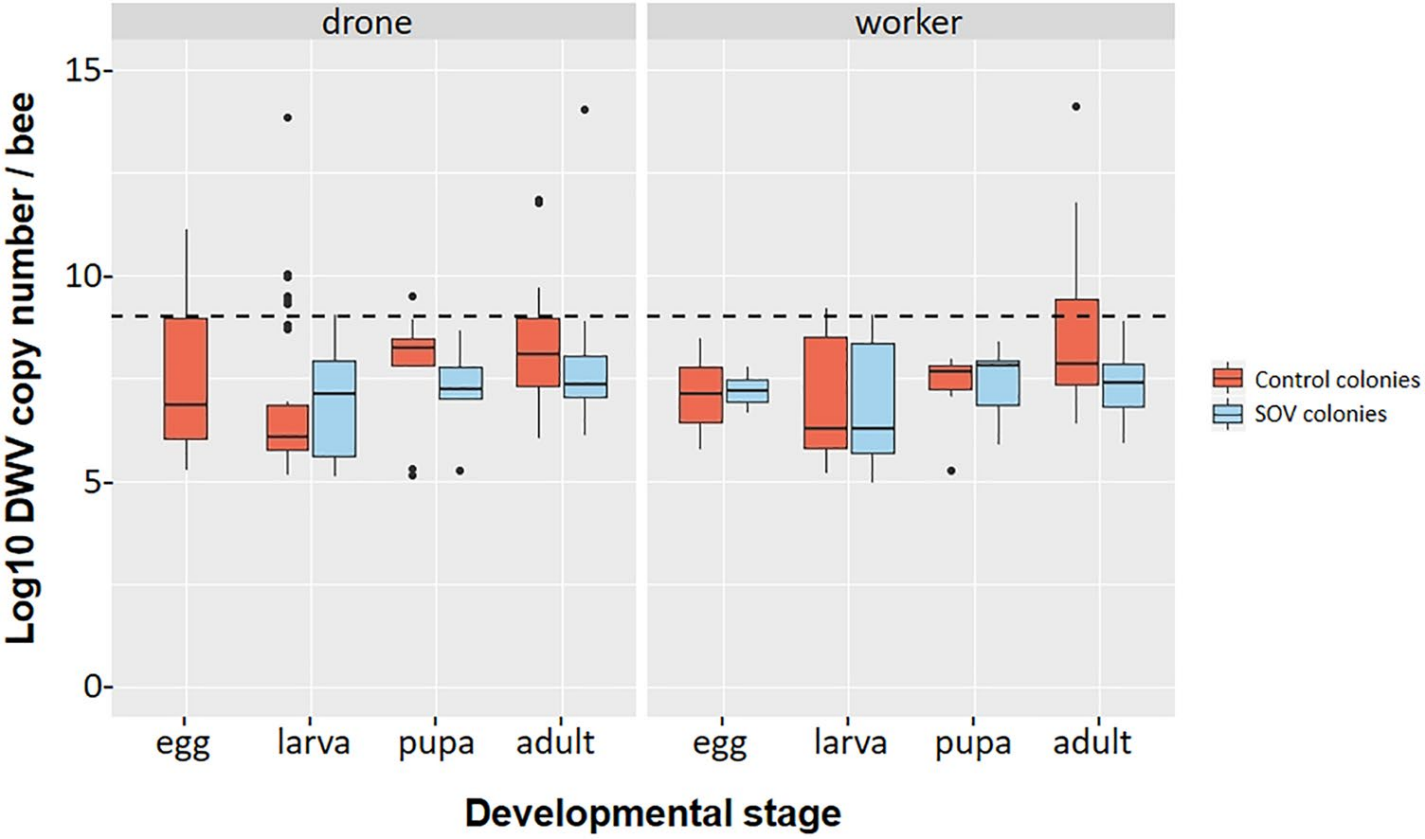
Box plot of DWV-load expressed in log10 DWV copy number/bee (or log10 DWV copy number/10 pooled eggs) across different developmental stages of drones (left) and worker bees (right). The horizontal dashed line represents the threshold of severe DWV infections (> 10^9^ viral copes per bee). Data on drone eggs of the SOV colonies are not shown in the graph as all samples were below the limit of detection of the qRT-PCR.

We subsequently determined the impact of the SOV trait on the DWV and SBV infection levels of four developmental stages of drones and workers of 4 SOV colonies (SOV group) in comparison to 4 control colonies (control group). The colonies were kept at 4 apiaries, with 1 SOV colony and 1 control colony each. Due to the extremely low SBV infection levels, these results were not informative (see Supplementary Table S2 online). The DWV infection levels, on the other hand, allowed comparison between the two groups (see Supplementary Table S3 online). With the threshold value for a positive virus determination set at 10^5^ viral genomic copies per sample, we saw a strong beneficial effect of the SOV trait on the male bee caste with fewer infections overall [Chi-squared (1, *N* = 245) = 12.4, *p* < 0.01] and for the larva [Chi-squared (1, *N* = 77) = 7.5, *p* < 0.01] and pupa developmental stage [Chi-squared (1, *N* = 80) = 5.7, *p* < 0.05; Fig. 3A]. Drone eggs did not significantly differ (Fisher’s exact test, *p* = 0.07) although being considerably lower with three infected samples in the control group to no infected samples in the SOV positive group. This non-significance is probably due to the low sample size due to pooling of the eggs. In the female caste, these differences were less pronounced and limited to adult bees [Chi-squared (1, *N* = 80) = 7.3, *p* < 0.01; Fig. 3B]. In contrast, worker pupa had a higher number of infected samples in the SOV positive group [Chi-squared (1, *N* = 80) = 5.2, *p* < 0.05]. Across all castes, 53% of the samples taken from SOV colonies were DWV positive, compared to 64% in the control group [Chi-squared (1, *N* = 493) = 6.7, *p* < 0.05]. The outcome was more pronounced with the threshold value set at 10^9^ viral genomic copies per sample, representing samples with a severe DWV load. We found that such harmful DWV infections hardly occur in the colonies selected for the SOV trait (1%), whereas in the control colonies they were found in 14% of the samples [Chi-squared (1, *N* = 493) = 27.7, *p* < 0.01]. The number of severe infections is significantly lower across all samples of workers and drones, and for the pupae and adults of the two castes [all worker samples: Chi-squared (1, *N* = 248) = 15.3, *p* < 0.01; worker pupae: Chi-squared (1, *N* = 80) = 5.3, *p* < 0.05; adult workers: Chi-squared (1, *N* = 80) = 14.1, *p* < 0.01; all drone samples: Chi-squared (1, *N* = 245) = 12.4, *p* < 0.01; drone pupae: Chi-squared (1, *N* = 80) = 4.2, *p* < 0.05; adult drones: Chi-squared (1, *N* = 80) = 8.5, *p* < 0.01; Fig. 3C,D]. These results matched with the observation that the virus loads in the SOV colonies (median of log10 DWV copy numbers per bee = 5.3) were significantly lower than those in the control colonies [= 6.0; one-way ANOVA; F (1, *N* = 286) = 4.648, *p* < 0.05; see also Fig. 4]. The effect of individual hives, where highly infected hives have an disproportional impact on the overall infection loads in the SOV group or control group, was not significant for the number of DWV infections (Mann Whitney test; U = 27,866, z = -1.069, *p* = 0.285). Together with the equal distribution of the SOV group and the control group between the apiaries we could assure that the trait rather than the environment or the circumstances at the apiary was here the determining factor.

## Discussion

The present study suggests for the first time that the honey bee’s potential to control virus infections is heritable. There are some observations of isolated *Varroa*-resistant honey bee populations with lowered viral loads or diminished clinical signs^28^, but none of these studies demonstrated a genetic cause. Our findings are based on the sanitary control of breeding queens conducted on the sideline of an ongoing breeding program. The estimated heritabilities were moderate and were all about 0.25. For comparison, traditional honey bee traits like honey yield, gentleness and calmness, but also those related to *Varroa* resistance/tolerance, have heritabilities which vary from 0.17 to 0.91 for the effect of workers and from 0.10–0.70 for the effect of queens^29–33^. Indeed, several traditional traits proved to be affected by the genotypes of workers and queens as well, while the novel trait described in this study obviously solely is affected by the queen. The fact that the estimated heritabilities of SOV described in the present study are of the same size of order justifies its implementation in breeding programs. Given the fact that testing for the SOV trait demands laboratory analyses and the instability of the viral RNA genome under ambient temperature, the implementation of this trait requires to set in place a cold chain for the egg samples. This could be part of a concerted action of the responsible organisation or (beekeepers) association.

We hypothesized that the virus status of the egg is a reflection of the health status—and by extension of the immune potential—of the queen, though we never verified this. As this study was interwoven in a running breeding program, in which breeders tested the offspring of their most precious queens, this was impossible to do. However, we did verify what the SOV trait means in terms of virus infections in the different developmental stages and castes of the colony. And the outcome was very promising: it seems that the honey bee egg holds the key of resilience to virus infection. The SOV trait provides no sterile immunity against viruses as they are still present, but some yet unknown mechanism seems to avoid that later exposures to—for instance—DWV will end with harmful infection levels.

The immunity of the queen against viruses certainly has also a social and behavioral component, but once a queen has acquired a virus infection, the propagation of the virus and the disease progression happens as in worker bees^34^. Bees like other insects have developed an antiviral immunity that relies on RNA interference (RNAi) among other pathways^35^. In an earlier study we found that the expression level of key components of the RNAi machinery are downregulated in heavily virus infected colonies^36^. We then suggested that viral RNAi suppressors mute the anti-viral immunity, a process that previously was described for the bees’ Israeli acute paralysis virus^37^. The differences in expression profiles could, however, also be explained as a functional RNAi response that keeps viruses under control in the subgroup with the lowest virus load. No such responses have been studied in honey bee queens yet, but it seems plausible that RNAi plays a role in the expression of the SOV trait. In *Caenorhabditis elegans* it has been demonstrated that the antiviral RNAi response not only inhibits vertical transmission, but also promotes transgenerational inheritance of antiviral immunity. Such heritable antiviral immunity seems to be mediated by small RNAs^38^. If similar mechanisms would occur in honey bees, the predisposition of an effective antiviral immunity of the queens could be inherited to the worker bees and protect the colony from virus related pathologies, as suggested by our in depth study of the virus load of four SOV colonies and their controls. Given the fact that viruses play a crucial role in the collapse of honey bee colonies, the implications could be tremendous. Moreover, if worker bees are capable of clearing a virus infection the overall virus contamination level of the colony will drop gradually, a process from which even bees of neighboring colonies that lack the protective genetic profile will benefit. Our data are suggesting this is happening as also in the DV + Q subgroup both the DWV and SBV infections dropped considerably over the last 2 years.

In conclusion, we discovered a new trait that renders honey bee eggs free of virus infection. We used the term ‘suppressed *in ovo* virus infection’ and demonstrated that the trait is heritable through the genotype of the queen, and not that of the worker, and can be expressed against several viruses simultaneously or against each of the viruses individually. The estimated heritability seems to be moderate with a value of about 0.25. The trait has a beneficial effect on the virus load of the colony as a whole with fewer and less severe DWV infections and its implementation into breeding programs is recommended.

## Materials and methods

### Sample collection

Flemish queen breeders provided between 1 and 45 samples each (see Supplementary Table S1 online). From each selected colony, 10 eggs from drone cells were pooled in a pre-labeled 1.5 ml tube. After collection the eggs were immediately stored at − 20 °C and transported to the laboratory maintaining the cold chain where they were stored at − 80 °C until they were processed for virus status determination. All samples were collected in June (from 2015 to 2018).

At the end of the study (between June, 20 and July, 15, 2019) and with the queens’ sanitary reports in hands, we selected 4 apiaries for an in depth study of the viral infection levels across different developmental stages and castes of the honey bee. The apiaries were managed by experienced beekeepers following standard beekeeping practices, including *Varroa* control. In each apiary one SOV colony and one control colony was selected based on the sanitary report of the queen in the previous year (2018) as determined by RT-PCR (see below) on a pooled egg sample. Of each colony 10 freshly laid eggs (pooled sample), 10 stretched larvae, 10 red-eyed pupae and 10 adults were collected for each caste (drones and workers). Where possible guard worker bees were selected in order to minimize the risk of sampling bees drifted from other colonies. Three drone larva samples could not be collected as one queen showed an irregular drone laying pattern and eventually ceased laying drone brood around the beginning of the sampling period. Placement of a drone brood frame in the hive to evoke the onset drone brood laying yielded insufficient success. Samples were kept in a cold chain as described here above.

### RNA extraction and cDNA synthesis

The eggs were homogenized in the presence of zirconium beads in 0.5 ml QIAzol lysis reagent (Qiagen). For larvae, pupae and adults 1 ml QIAzol lysis reagent was used. RNA was extracted using the RNeasy Lipid tissue mini kit (Qiagen) according to the manufactures instructions and finally eluted in 30 µl elution buffer. The concentration of the total RNA was measured with a Nanodrop (Isogen). Using random hexamer primers, 200 ng RNA was retro-transcribed with the RevertAid H Minus First Strand cDNA Synthesis Kit (Thermo Scientific).

### Virus analysis by RT-PCR

The eggs were examined by uniplex RT-PCR assays for the presence of viruses of the ABPV complex, BQCV, DWV and SBV. We used honey bee β-actin as a control gene. All PCR reaction mixtures contained: 2 μM of each primer (see Supplementary Table S4); 1 mM MgCl_2_; 0.2 mM dNTPs each; 1.2 U HotStarTaq Plus DNA polymerase (Qiagen) and 2 μl cDNA product. PCR assays were performed using the following cycling conditions: 95 °C—5 min; 94 °C—30 s, 55 °C—30 s, 72 °C—1 min, 35 cycles; final elongation 72 °C—10 min, hold 4 °C. PCR amplicons were separated by electrophoresis using 1.5% agarose gels stained with ethidium bromide and visualized under UV light.

### Virus analysis by qRT-PCR

The comparison of the virus load across developmental stages was done by a uniplex qRT-PCR or real-time PCR using Platinum SYBR Green qPCR SuperMix-UDG (Thermo Scientific). Each reaction consisted of 0.4 µM of each primer (see Supplementary Table S4), 11.45 µl RNase-free water, 12.5 µl SYBR Green and 1 µl of cDNA template. All samples were run in duplicate in a three-step real-time qPCR with following thermal cycling conditions: denaturation stage at 95 °C—15 s, annealing stage at 58 °C—20 s and extension stage at 72 °C—30 s for 35 cycles. This procedure was followed by a melt-curve dissociation analysis to confirm the specificity of the product (55–95 °C with an increment of 0.5 °C s^−1^). Each plate included a no target control (NTC). Viral loads of SBV and DWV were quantified using absolute quantification methods based on a standard curve obtained through a eightfold 5 × serial dilution of viral plasmid loads that ranged between 10^4^–10^10^ copies/µl. All data were analyzed using CFX Manager 3.1 software (Bio-Rad). Baseline correction and threshold setting were performed using the automatic calculation offered by the same software. Maximum accepted quantification cycle (Ct) difference between replicates was set to one Ct. The successful amplification of the reference gene β-actin was used to confirm the integrity of samples throughout the entire procedure.

### Quantitative genetic analysis

The purpose of the quantitative genetic analysis was to estimate heritabilities, to look at the environmental similarity of virus scores of queens and their dams, and to estimate correlations between the scores of different viruses. Because for this analysis the genetic relationships between queens are required the analysis was restricted to the records of 560 Carnica queens. This included 176 queens without a pedigree, but of the 384 other queens the dams were known and in 58% of the cases also the sires. These sires were drone-producing queens on mating islands (54% of the cases) or used to produce drones for instrumental insemination. The pedigrees were traced back to 2005 using historical information in the data base www.beebreed.eu. Beebreed is an international data base that annually grows with some 10,000 queens with pedigrees and breeding values for traits like honey yield, gentleness and varroa-resistance. The extended pedigree was used to derive the additive genetic relationships matrix between individuals in the pedigree to allow estimation of heritabilities^39^.

The linear animal model describing the observations was as in Eq. (1).

1 $${y}_{ijk}=\mu +{q}_{i}+{vd}_{j}+{t}_{k}+{e}_{ijk}$$

In this model

- *y*_*ik*_ stands for the observation (SOV/ABPV, SOV/BQCV, SOV/DWV, SOV/SBV or SOV/TVS);
- $\mu$ stands for the overall mean;
- ${q}_{i}$ is a random effect representing the breeding value of the ${i}\text{th}$ queen producing the drone eggs, giving an estimate for the additive genetic variance component ${\sigma }_{q}^{2}$;
- ${vd}_{j}$ is a fixed effect for the jth virus-score class of the dam. There are three of those classes: unknown, no virus present and virus present;
- ${t}_{k}$ is a fixed effect for the *k*th test location in which an apiary in another year is considered to be a different test location. There were 101 test locations with 1 to 18 records each;
- ${e}_{ijk}$ is a random residual term, giving a variance component for residual environmental effect ${\sigma }_{e}^{2}$.

The presence of both ${q}_{i}$ and ${vd}_{j}$ in the model enables to distinguish between genetic causes that the virus scores of queens resemble those of their dams on the one hand and non-genetic causes (environmental similarity) on the other hand. In theory, a model not including ${vd}_{j}$ might lead to overestimation of ${\sigma }_{q}^{2}$ and consequently of the heritability.

Although in the model all effects were estimated simultaneously, one might say that all queens with observations contributed to estimate the effect of test location, that cases where queen and dam have an observation contributed to the estimation of environmental similarity and that the genetic relationships between queens allowed to estimate ${\sigma }_{q}^{2}$.

The statistical analyses were carried out using ASReml^40^. We analysed the models assuming a binomial distribution of the phenotypes (Table 3) with an underlying normally-distributed genetic liability linked to the observed scale by a logit function. A binomial distribution seemed a logical choice as the scores were 0 or 1, and the low incidence makes a linear model not suited.

**Table 3:** Distribution of the data of the 560 Carnica queens. There were 16 queens that provided two samples and one queen with three samples. The virus score for those cases was the average of those of the different samples. The scores for TVS were the sum of those of the individual viruses. The results in Tables 1 and 2 resulted from analyses in which the presence of viruses was scored 0 or 1, where all scores larger than 0 are put to 1.

	Occasion of viruses						
	0	0.5	1	1.5	2	3	4
ABPV	519	3	38				
BQCV	454	1	105				
DWV	449	4	107				
SBV	477	3	80				
TVS	320	4	162	1	52	18	3

### Heritability estimates

Heritabilities for the different traits were estimated according to Eq. (2).

2 $${h}^{2}=\frac{{\sigma }_{q}^{2}}{{\sigma }_{q}^{2}+{\sigma }_{e}^{2}}$$

where variance components ${\sigma }_{q}^{2}$ were the result of the analyses and the implicit residual variance ${\sigma }_{e}^{2}$ for the underlying logistic distribution was $\frac{{\pi }^{2}}{3}\approx 3.3$ for the logit link function^40^.

### Correlations

The dataset was too small to estimate genetic correlations. Conceptually for each observation the deviation was computed from the estimate of the fixed effect of test location and pairwise Pearson correlations were computed between those deviations. The correlations and their standard errors were estimated using ASReml^40^ with a model that only included the overall mean, the effect of test location and the random residual effect.

### Statistics on qRT-PCR data

To improve data compliance with statistical tests and data visualization, viral loads of all samples were log10 transformed. Data analysis and visualization were performed using ‘R’ version 3.6.1. The level of 10^9^ DWV genomic copies per bee was used as a threshold to distinguish ‘severely infected bees’. This threshold is reported in multiple studies as the transition between covert to overt infection^41–43^. For analyses of the differences in the number of DWV infections and severe DWV infections the Chi-squared test or the Fisher’s exact test was used depending on the sample size. Differences in infection loads between groups were analyzed by one-way ANOVA. A possible disproportional effect of individual hives on the number of infections was analyzed using the Mann–Whitney test.

## Supplementary information

Supplementary Information.

## Data Availability

Rough and trimmed data of honey bee breeding for suppressed *in ovo* virus infection, together with data on the kinship of the tested queens (pedigree) generated during the current study are available in the figshare repository: https://doi.org/10.6084/m9.figshare.8170925.
