# Supplementary material for: Heritability estimates of the novel trait ‘suppressed in ovo virus infection’ in honey bees (Apis mellifera)
Source: Sci Rep. 2020 Aug 31;10:14310. doi: 10.1038/s41598-020-71388-x (PMC7459113; doi:10.1038/s41598-020-71388-x)
Supplement: Supplementary file 1 — Supplementary Information. [file 41598_2020_71388_MOESM1_ESM.docx]

**Supplementary Information**

**Heritability estimates of the novel trait ‘suppressed *in ovo* virus infection’ in honey bees (*Apis mellifera*)**

Dirk C. de Graaf^1,2^, Dries Laget^1^, Lina De Smet^2^, David Claeys Boúúaert^2^, Marleen Brunain^1,2^, Roel F. Veerkamp^3^, Evert W. Brascamp^3^

^1^Honeybee Valley, Ghent University, B-9000, Ghent, Belgium. ^2^Department of Biochemistry and Microbiology, Ghent University, B-9000, Ghent, Belgium. ^3^Animal Breeding & Genomics, Wageningen University and Research, 6708 PB, Wageningen, The Netherlands.

**Supplementary Figure S1.** Progress of the selection work over time. (A) Participation rate. Number of egg samples analyzed per year for the sanitary control of breeding queens. Samples were categorized in three defined subgroups: descendants of virus positive queens (DV+Q), descendants of virus negative queens (DV-Q) and unspecified daughter queens (UQ). Numbers of samples examined of each subgroup are printed in the corresponding boxes. (B) Percentage of samples with a positive total virus status (TVS).

**A**


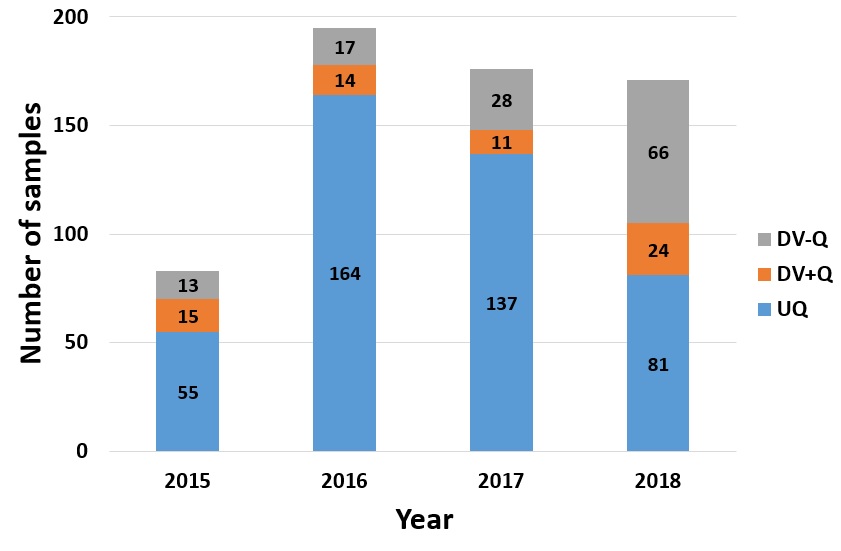


**B**

**
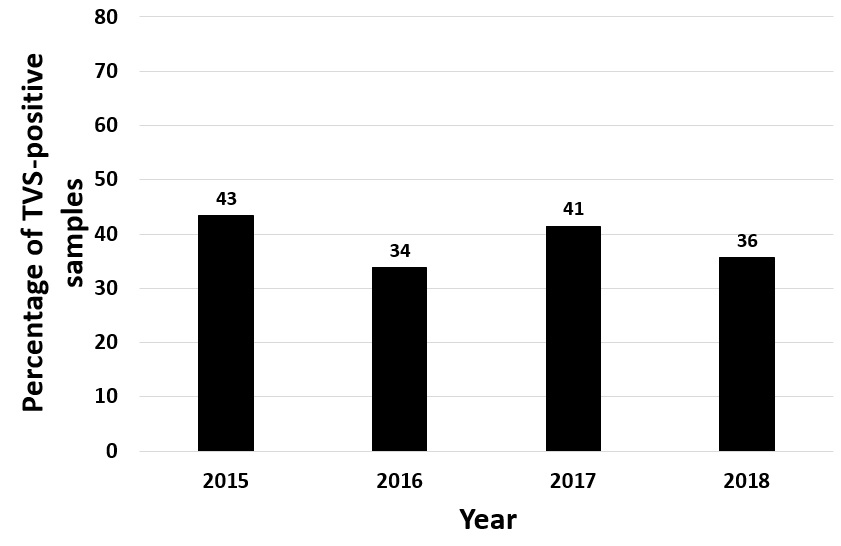
**

**Supplementary Table S1.** Overview of the sampling taken in the context of the sanitary control of breeding queens. Number of tested queens per beekeeper for each year are given, with indication of the *Apis mellifera* subspecies or strain involved.

| **Beekeeper code** | **Subspecies**  **/ Strain** | **Sanitary controlled queens** | | | | |
| --- | --- | --- | --- | --- | --- | --- |
|  |  | **2015** | **2016** | **2017** | **2018** | **Total** |
| ABO | Unknown |  |  | 3 |  | **3** |
| ADC | Carnica | 4 | 10 |  | 6 | **20** |
| ADH | Buckfast | 6 | 5 |  | 5 | **16** |
| ARE | Carnica |  | 6 |  |  | **6** |
| ARH | Carnica | 5 | 7 | 6 | 10 | **28** |
| AVE | Carnica |  | 1 | 6 | 10 | **17** |
| AVV | Carnica |  | 2 | 2 | 6 | **10** |
| BDS | Carnica |  | 2 |  |  | **2** |
| DCR | Carnica |  | 5 |  |  | **5** |
| DVD | Carnica | 18 |  | 7 |  | **25** |
| DVK | Carnica |  | 10 | 12 | 12 | **34** |
| DVT | Carnica |  | 1 |  |  | **1** |
| DVU | Carnica |  | 2 |  |  | **2** |
| EMA | Carnica |  | 10 |  |  | **10** |
| FDC | Carnica | 2 | 10 |  |  | **12** |
| FIM | Carnica |  |  | 2 |  | **2** |
| FVD | Carnica |  | 6 | 9 | 3 | **18** |
| GHD | Carnica | 8 | 10 | 9 | 18 | **45** |
| GLO | Carnica |  | 1 | 1 |  | **2** |
| GSV | Carnica | 7 |  | 1 | 4 | **12** |
| GVD | Carnica |  | 2 |  |  | **2** |
| GVE | Unknown |  | 5 |  |  | **5** |
| HBV | Carnica |  |  | 8 | 2 | **10** |
| HDA | Unknown |  | 8 |  |  | **8** |
| HEP | Carnica | 3 | 6 | 8 | 10 | **27** |
| HSC | Carnica |  | 4 |  |  | **4** |
| HSE | Carnica |  | 3 |  |  | **3** |
| HSW | Carnica | 3 | 3 | 2 |  | **8** |
| JDR | Carnica |  |  | 2 |  | **2** |
| JDR | Buckfast |  |  |  | 4 | **4** |
| JDS | Carnica | 5 | 2 | 12 | 1 | **20** |
| JJE | Carnica | 2 |  | 5 |  | **7** |
| JMA | Carnica |  | 10 | 9 | 6 | **25** |
| JRU | Carnica |  |  | 2 |  | **2** |
| KDW | Carnica |  | 4 | 1 | 2 | **7** |
| LMO | Unknown |  | 1 |  |  | **1** |
| MKH | Carnica |  |  | 12 | 12 | **24** |
| MME | Unknown |  | 6 |  |  | **6** |
| MME | Carnica |  |  | 1 |  | **1** |
| OVL | Carnica |  |  | 12 | 12 | **24** |
| PBU | Carnica |  |  |  | 1 | **1** |
| PDU | Carnica |  | 2 | 5 | 5 | **12** |
| PGO | Buckfast |  | 2 |  |  | **2** |
| RBO | Carnica |  | 2 |  |  | **2** |
| RDV | Carnica | 9 | 9 | 6 | 6 | **30** |
| RGD | Buckfast |  | 2 | 1 |  | **3** |
| RLA | Carnica |  | 3 | 6 | 9 | **18** |
| RRV | Carnica | 3 | 10 | 5 | 5 | **23** |
| RTA | Carnica |  |  | 4 | 4 | **8** |
| RVV | Carnica |  |  | 2 |  | **2** |
| SKA | Carnica |  |  | 2 |  | **2** |
| TJA | Carnica |  | 10 | 3 | 7 | **20** |
| WDR | Carnica | 4 | 6 | 10 | 11 | **31** |
| WGR | Carnica |  | 2 |  |  | **2** |
| WHU | Carnica |  | 2 |  |  | **2** |
| WRA | Carnica | 4 | 3 |  |  | **7** |
| **Total** |  | **83** | **195** | **176** | **171** | **625** |

**Supplementary Table S2.** Overview of the SBV load of SOV and control colonies.

|  |  | **Number of SBV infected samples** | | | | | | **Number of samples with severe SBV infection** | | | | | | **Average SBV infection (Log10)** | | | | |
| --- | --- | --- | --- | --- | --- | --- | --- | --- | --- | --- | --- | --- | --- | --- | --- | --- | --- | --- |
|  |  | egg | larva | pupa | adult | Total | Percentage | egg | larva | pupa | adult | Total | Percentage | egg | larva | pupa | adult | Total |
| **Control colonies** | **drone** | **0/4** | **1/40** | **5/40** | **3/40** | **9/124** | **7%** | **0/4** | **0/40** | **0/40** | **0/40** | **0/124** | **0%** | **ND** | **5,3** | **5,3** | **5,3** | **5,3** |
|  | Apiary 1 - hive 1 | 0/1 | 0/10 | 0/10 | 0/10 | 0/31 | 0% | 0/1 | 0/10 | 0/10 | 0/10 | 0/31 | 0% | ND | ND | ND | ND | ND |
|  | Apiary 2 - hive 2 | 0/1 | 0/10 | 0/10 | 0/10 | 0/31 | 0% | 0/1 | 0/10 | 0/10 | 0/10 | 0/31 | 0% | ND | ND | ND | ND | ND |
|  | Apiary 3 - hive 3 | 0/1 | 1/10 | 5/10 | 3/10 | 9/31 | 29% | 0/1 | 0/10 | 0/10 | 0/10 | 0/31 | 0% | ND | **5,3** | **5,3** | **5,3** | **5,3** |
|  | Apiary 4 - hive 4 | 0/1 | 0/10 | 0/10 | 0/10 | 0/31 | 0% | 0/1 | 0/10 | 0/10 | 0/10 | 0/31 | 0% | ND | ND | ND | ND | ND |
|  | **worker** | **0/4** | **1/40** | **3/40** | **2/40** | **6/124** | **5%** | **0/4** | **0/40** | **0/40** | **0/40** | **0/124** | **0** | **ND** | **5,3** | **5,2** | **5,1** | **5,2** |
|  | Apiary 1 - hive 1 | 0/1 | 0/10 | 0/10 | 0/10 | 0/31 | 0% | 0/1 | 0/10 | 0/10 | 0/10 | 0/31 | 0% | ND | ND | ND | ND | ND |
|  | Apiary 2 - hive 2 | 0/1 | 0/10 | 0/10 | 0/10 | 0/31 | 0% | 0/1 | 0/10 | 0/10 | 0/10 | 0/31 | 0% | ND | ND | ND | ND | ND |
|  | Apiary 3 - hive 3 | 0/1 | 1/10 | 3/10 | 2/10 | 6/31 | 19% | 0/1 | 0/10 | 0/10 | 0/10 | 0/31 | 0% | ND | 5,3 | 5,2 | 5,1 | 5,2 |
|  | Apiary 4 - hive 4 | 0/1 | 0/10 | 0/10 | 0/10 | 0/31 | 0% | 0/1 | 0/10 | 0/10 | 0/10 | 0/31 | 0% | ND | ND | ND | ND | ND |
|  | **Total** | **0/8** | **2/80** | **8/80** | **5/80** | **0/248** | **0** | **0/8** | **0/80** | **0/80** | **0/80** | **0/248** | **0%** | **ND** | **5,3** | **5,2** | **5,1** | **5,2** |
| **SOV colonies** | **drone** | **0/4** | **0/37** | **0/40** | **0/40** | **0/121** | **0** | **0/4** | **0/37** | **0/40** | **0/40** | **0/121** | **0%** | **ND** | **ND** | **ND** | **ND** | **ND** |
|  | Apiary 1 - hive 5 | 0/1 | 0/7 | 0/10 | 0/10 | 0/28 | 0% | 0/1 | 0/7 | 0/10 | 0/10 | 0/28 | 0% | ND | ND | ND | ND | ND |
|  | Apiary 2 - hive 6 | 0/1 | 0/10 | 0/10 | 0/10 | 0/31 | 0% | 0/1 | 0/10 | 0/10 | 0/10 | 0/31 | 0% | ND | ND | ND | ND | ND |
|  | Apiary 3 - hive 7 | 0/1 | 0/10 | 0/10 | 0/10 | 0/31 | 0% | 0/1 | 0/10 | 0/10 | 0/10 | 0/31 | 0% | ND | ND | ND | ND | ND |
|  | Apiary 4 - hive 8 | 0/1 | 0/10 | 0/10 | 0/10 | 0/31 | 0% | 0/1 | 0/10 | 0/10 | 0/10 | 0/31 | 0% | ND | ND | ND | ND | ND |
|  | **worker** | **0/4** | **1/40** | **3/40** | **1/40** | **5/124** | **4%** | **0/4** | **0/40** | **0/40** | **0/40** | **0/124** | **0** | **ND** | **4,3** | **4,6** | **ND** | **4,5** |
|  | Apiary 1 - hive 5 | 0/1 | 0/10 | 0/10 | 0/10 | 0/31 | 0% | 0/1 | 0/10 | 0/10 | 0/10 | 0/31 | 0% | ND | ND | ND | ND | ND |
|  | Apiary 2 - hive 6 | 0/1 | 0/10 | 0/10 | 0/10 | 0/31 | 0% | 0/1 | 0/10 | 0/10 | 0/10 | 0/31 | 0% | ND | ND | ND | ND | ND |
|  | Apiary 3 - hive 7 | 0/1 | 0/10 | 0/10 | 0/10 | 0/31 | 0% | 0/1 | 0/10 | 0/10 | 0/10 | 0/31 | 0% | ND | ND | ND | ND | ND |
|  | Apiary 4 - hive 8 | 0/1 | 1/10 | 3/10 | 1/10 | 5/31 | 16% | 0/1 | 0/10 | 0/10 | 0/10 | 0/31 | 0% | ND | 4,3 | 4,6 | ND | 4,5 |
|  | **Total** | **0/8** | **1/77** | **3/80** | **1/80** | **5/245** | **2%** | **0/8** | **0/77** | **0/80** | **0/80** | **0/245** | **0%** | **ND** | **4,3** | **4,6** | **ND** | **4,5** |

**Supplementary Table S3.** Overview of the DWV load of SOV and control colonies.

|  |  | **Number of DWV infected samples** | | | | | | **Number of samples with severe DWV infection** | | | | | | **Average DWV infection (Log10)** | | | | |
| --- | --- | --- | --- | --- | --- | --- | --- | --- | --- | --- | --- | --- | --- | --- | --- | --- | --- | --- |
|  |  | egg | larva | pupa | adult | Total | Percentage | egg | larva | pupa | adult | Total | Percentage | egg | larva | pupa | adult | Total |
| **Control colonies** | **drone** | **3/4** | **34/40** | **18/40** | **29/40** | **84/124** | **68%** | **1/4** | **2/40** | **4/40** | **10/40** | **16/124** | **13%** | **8,4** | **7,2** | **7,7** | **8,5** | **7,79** |
|  | Apiary 1 - hive 1 | 0/1 | 7/10 | 2/10 | 4/10 | 13/31 | 42% | 0/1 | 0/10 | 0/10 | 1/10 | 1/31 | 3% | ND | 6,2 | 5,1 | 8,7 | 6,93 |
|  | Apiary 2 - hive 2 | 1/1 | 8/10 | 8/10 | 10/10 | 27/31 | 87% | 1/1 | 1/10 | 2/10 | 4/10 | 8/31 | 26% | 11,1 | 7,6 | 8,4 | 8,7 | 8,41 |
|  | Apiary 3 - hive 3 | 1/1 | 9/10 | 2/10 | 7/10 | 19/31 | 61% | 0/1 | 0/10 | 0/10 | 2/10 | 1/31 | 3% | 5,8 | 6,1 | 6,1 | 8,1 | 6,83 |
|  | Apiary 4 - hive 4 | 1/1 | 10/10 | 6/10 | 8/10 | 25/31 | 81% | 0/1 | 1/10 | 2/10 | 3/10 | 6/31 | 19% | 8,4 | 8,4 | 7,8 | 8,2 | 8,25 |
|  | **worker** | **2/4** | **28/40** | **6/40** | **39/40** | **75/124** | **60%** | **0/4** | **0/40** | **5/40** | **12/40** | **18/124** | **15%** | **6,0** | **6,6** | **6,4** | **8,4** | **7,53** |
|  | Apiary 1 - hive 1 | 1/1 | 10/10 | 2/10 | 9/10 | 22/31 | 71% | 0/1 | 0/10 | 1/10 | 2/10 | 3/31 | 10% | 6,8 | 5,8 | ND | 8,7 | 7,07 |
|  | Apiary 2 - hive 2 | 1/1 | 10/10 | 1/10 | 10/10 | 22/31 | 71% | 0/1 | 0/10 | 1/10 | 0/10 | 1/31 | 3% | 5,3 | 8,0 | 5,3 | 6,9 | 7,28 |
|  | Apiary 3 - hive 3 | 0/1 | 2/10 | 2/10 | 10/10 | 14/31 | 45% | 0/1 | 0/10 | 2/10 | 3/10 | 5/31 | 16% | ND | 5,8 | ND | 8,2 | 7,89 |
|  | Apiary 4 - hive 4 | 0/1 | 6/10 | 1/10 | 10/10 | 17/31 | 55% | 0/1 | 0/10 | 1/10 | 7/10 | 8/31 | 25% | ND | 5,8 | 7,4 | 9,3 | 8,10 |
|  | **Total** | **5/8** | **62/80** | **24/80** | **68/80** | **159/248** | **64%** | **1/8** | **2/80** | **9/80** | **22/80** | **34/248** | **14%** | **7,5** | **6,9** | **7,6** | **8,4** | **7,67** |
| **SOV colonies** | **drone** | **0/4** | **21/37** | **8/40** | **26/40** | **55/121** | **45%** | **0/4** | **1/37** | **0/40** | **1/40** | **2/121** | **2%** |  | **6,3** | **6,4** | **7,5** | **6,91** |
|  | Apiary 1 - hive 5 | 0/1 | 3/7 | 0/10 | 9/10 | 12/28 | 39% | 0/1 | 0/7 | 0/10 | 1/10 | 1/28 | 3% | ND | 7,0 | ND | 7,7 | 7,52 |
|  | Apiary 2 - hive 6 | 0/1 | 4/10 | 1/10 | 4/10 | 9/31 | 29% | 0/1 | 1/10 | 0/10 | 0/10 | 1/31 | 3% | ND | 7,7 | 5,2 | 7,8 | 7,44 |
|  | Apiary 3 - hive 7 | 0/1 | 5/10 | 3/10 | 8/10 | 16/31 | 57% | 0/1 | 0/10 | 0/10 | 0/10 | 0/31 | 0% | ND | 5,4 | 5,9 | 7,3 | 6,42 |
|  | Apiary 4 - hive 8 | 0/1 | 9/10 | 4/10 | 5/10 | 18/31 | 58% | 0/1 | 0/10 | 0/10 | 0/10 | 0/31 | 0% | ND | 6,0 | 7,5 | 7,4 | 6,59 |
|  | **worker** | **2/4** | **26/40** | **15/40** | **31/40** | **74/124** | **60%** | **0/4** | **1/40** | **0/40** | **0/40** | **1/124** | **1%** | **7,2** | **7,4** | **7,6** | **7,5** | **7,46** |
|  | Apiary 1 - hive 5 | 0/1 | 2/10 | 2/10 | 9/10 | 13/31 | 42% | 0/1 | 0/10 | 0/10 | 0/10 | 0/31 | 0% | ND | 5,4 | 7,4 | 7,5 | 7,14 |
|  | Apiary 2 - hive 6 | 0/1 | 9/10 | 3/10 | 7/10 | 19/31 | 61% | 0/1 | 0/10 | 0/10 | 0/10 | 0/31 | 0% | ND | 6,9 | 7,7 | 7,8 | 7,37 |
|  | Apiary 3 - hive 7 | 1/1 | 6/10 | 1/10 | 9/10 | 17/31 | 55% | 0/1 | 0/10 | 0/10 | 0/10 | 0/31 | 0% | 7,8 | 6,9 | 5,9 | 7,4 | 7,19 |
|  | Apiary 4 - hive 8 | 1/1 | 9/10 | 9/10 | 6/10 | 25/31 | 81% | 0/1 | 1/10 | 0/10 | 0/10 | 1/31 | 3% | 6,7 | 8,6 | 7,9 | 7,2 | 7,91 |
|  | **Total** | **2/8** | **47/77** | **23/80** | **57/80** | **129/245** | **53%** | **0/8** | **2/77** | **0/80** | **1/80** | **3/245** | **1%** | **7,2** | **6,9** | **7,3** | **7,5** | **7,24** |

**Supplementary Table S4. Sequence of the used primers in RT-PCR/qRT-PCR.**

| **Target** | **PCR-type** | **Sequence (5’ -> 3’)** | **References** |
| --- | --- | --- | --- |
| ABPV_F | RT-PCR | tcatacctgccgatcaag | ^1^ |
| ABPV_R | RT-PCR | ctgaataatactgtgcgtatc | ^1^ |
| BQCV_F | RT-PCR | agtggcggagatgtatgc | ^2^ |
| BQCV_R | RT-PCR | ggaggtgaagtggctatatc | ^2^ |
| DWV_F | RT-PCR | Ttcattaaagccacctggaacatc | ^3^ |
| DWV_R | RT-PCR | tttcctcattaactgtgtcgttga | ^3^ |
| DWV_F | qRT-PCR | ggtaagcgatggttgtttg | ^4^ |
| DVW_R | qRT-PCR | ccgtgaatatagtgtgagg | ^4^ |
| SBV_F  SBV_R | RT-PCR/qRT-PCR  RT-PCR/qRT-PCR | gctctaacctcgcatcaac  ttggaactacgcattctctg | ^2^  ^2^ |
| β-actin_F | RT-PCR | cgtgccgatagtattcttg | ^5^ |
| β-actin_R | RT-PCR | cttcgtcaccaacatagg | ^5^ |

**References**

1 Francis, R. M. & Kryger, P. Single Assay Detection of Acute Bee Paralysis Virus, Kashmir Bee Virus and Israeli Acute Paralysis Virus. *J Apic Sci* **56**, 137-146, doi:10.2478/v10289-012-0014-x (2012).

2 Singh, R. *et al.* RNA Viruses in Hymenopteran Pollinators: Evidence of Inter-Taxa Virus Transmission via Pollen and Potential Impact on Non-Apis Hymenopteran Species. *Plos One* **5**, e14357, doi:10.1371/journal.pone.0014357 (2010).

3 Forsgren, E., de Miranda, J. R., Isaksson, M., Wei, S. & Fries, I. Deformed wing virus associated with Tropilaelaps mercedesae infesting European honey bees (Apis mellifera). *Exp Appl Acarol* **47**, 87-97, doi:10.1007/s10493-008-9204-4 (2009).

4 Mondet, F. *et al.* Specific Cues Associated With Honey Bee Social Defence against Varroa destructor Infested Brood. *Sci Rep-Uk* **6**, 25444, doi:10.1038/srep25444 (2016).

5 Scharlaken, B., de Graaf, D. C., Goossens, K., Peelman, L. J. & Jacobs, F. J. Differential gene expression in the honeybee head after a bacteria challenge. *Dev Comp Immunol* **32**, 883-889, doi:10.1016/j.dci.2008.01.010 (2008).
